# 2035. Detecting Vancomycin-resistant Enterococci infection in an immunosuppressed population after screening

**DOI:** 10.1093/ofid/ofac492.1657

**Published:** 2022-12-15

**Authors:** Yasir Hamad, Nehal G Hashem, David K Henderson, Brooke K Decker, Robin T Odom

**Affiliations:** NIH Clinical Center, Bethesda, MD; NIH, Bethesda, Maryland; NIH, Bethesda, Maryland; National Institutes of Health, Bethesda, Maryland; NIH Clinical Center, Bethesda, MD

## Abstract

**Background:**

Vancomycin-Resistant Enterococci (VRE) are major pathogens for severely immunosuppressed patients. Because VRE are common pathogens in our institution, such patients are often empirically treated to cover VRE, exposing them to broad-spectrum antimicrobials. Analogous to nasal screening for Methicillin-resistant *Staphylococcus aureus* carriage, targeted VRE surveillance is performed to identify and isolate colonized patients to reduce transmission. To assess risks and benefits of empiric therapy, we evaluated this surveillance procedure.

**Methods:**

All patients admitted to two inpatient hospital units from 01/01/2019 to 12/31/2021 had routine perirectal VRE surveillance ordered and results retrospectively reviewed. One swab from each Culturette set was tested by *vanA* PCR, and the other was inoculated on CHROMEID® VRE (Biomerieux) if the PCR was positive. Subsequent clinical cultures of any site within 14 days of surveillance screening were reviewed for VRE.

**Results:**

1133 patients were screened; 46 (4.1%) patients had VRE colonization. Within 2 weeks following VRE screening 20 patients had clinical samples that were positive for an Enterococcus species (14 in the first 7 days and 6 in days 8-14). The most common sites of clinical cultures were urine, 8 (40%), wound, 4 (20%), intra-abdominal, 4 (20%), and blood, 2 (10%). No clinical culture sample contained VRE.

**Conclusion:**

The rate of VRE carriage was low in this population, raising the question of the value of broad-spectrum empiric therapy coverage for VRE. No VRE infections were subsequently detected in any patients irrespective of surveillance. The negative predictive value for clinical VRE infection was 100% with negative surveillance screening in this population.

**Disclosures:**

**All Authors**: No reported disclosures.

